# Real-world effectiveness and safety of difelikefalin for chronic kidney disease-associated pruritus: a systematic review

**DOI:** 10.1080/0886022X.2026.2711185

**Published:** 2026-08-11

**Authors:** Rocco Sheldon, Morya Wadodkar, Andrew Gan, Hung-Yeh Chien, Jyoti Baharani

**Affiliations:** ^a^ College of Medicine and Health, University of Birmingham, Birmingham, UK; ^b^ Bristol Medical School, University of Bristol, Bristol, UK; ^c^ Department of Renal Medicine, University Hospitals Birmingham NHS Foundation Trust, Birmingham, UK

**Keywords:** Difelikefalin, chronic kidney disease, pruritus, hemodialysis, dialysis

## Abstract

**Introduction:**

Chronic kidney disease-associated pruritus (CKD-aP) affects 55% of hemodialysis patients and substantially impairs quality of life (QoL) and treatment adherence. Difelikefalin, a selective kappa-opioid receptor agonist, received regulatory approval following the KALM trials. However, the applicability of these findings to routine practice remains unclear. We systematically evaluated real-world evidence (RWE) on the effectiveness and safety of difelikefalin in patients with CKD-aP.

**Methods:**

Comprehensive searches of PubMed, Embase, CENTRAL, Scopus, Web of Science, and CINAHL were conducted on 15 December 2025. Inclusion criteria were RWE studies, including observational studies and trial extension analyses reporting the efficacy (WI-NRS and 5-D Itch scores), QoL (Skindex-10), or safety in patients with CKD-aP using difelikefalin. Exclusion criteria included RCTs without open-label extensions and pruritus due to alternative causes. Two reviewers independently screened studies and extracted data. The study was prospectively registered as CRD420251266218.

**Results:**

Twelve studies (*n* = 3,524) met eligibility criteria. Across real-world settings, 35–92.9% of patients achieved *a* ≥ 3-point WI-NRS reduction, compared with 51% in pooled KALM-1/KALM-2 data. All studies assessing sleep-related QoL reported significant benefit. Overall AE incidence ranged from 0% to 64.4%, with 6.3–16% considered treatment-related. Common AEs included dizziness, diarrhea, nausea, hyperkalaemia, somnolence, and altered mental status. No treatment-related deaths or fatal serious adverse events were reported.

**Conclusions:**

RWE supports the effectiveness of difelikefalin in hemodialysis patients with CKD-aP. However, included studies are heterogeneous and at high risk of bias. Future research should investigate difelikefalin in peritoneal dialysis and earlier-stage CKD cohorts, alongside health economic evaluations and implementation studies.

## Introduction

### Background

Chronic kidney disease-associated pruritus (CKD-aP) is a distressing dermatological condition affecting patients with chronic kidney disease (CKD). It is characterized by itching directly related to kidney disease that is not explained by any other cause or primary skin lesion [1].

A 2018 meta-analysis reported the prevalence of CKD-aP at 55%, with similar rates across sex and dialysis types [2]. CKD-aP significantly impacts patients’ quality of life (QoL), affecting aspects such as sleep quality, anxiety, and depression [3–5]. Additionally, severe CKD-aP is associated with decreased hemodialysis (HD) compliance, increased medication usage, and higher hospitalization rates [4].

CKD-aP has traditionally been an underreported and underrecognised complication. 17% of patients who are nearly always or always bothered by CKD-aP do not report their symptoms to healthcare staff [6], and nephrologists report a perceived prevalence of CKD-aP of 43.6% [7], markedly lower than 55% identified by meta-analyses [2].

The pathogenesis of CKD-aP is poorly understood. Therefore, heterogenous unlicensed medications are used to targeting the various proposed mechanisms [6], including optimizing dialysis, emollients/moisturizers, oral antihistamines, gabapentin, ultraviolet light therapy, and antidepressants [7,8]. However, for all these treatments, queries remain about effectiveness and adverse event (AE) profiles [9].

Difelikefalin, a selective kappa-opioid receptor (KOR) agonist, became the first medication approved to treat moderate-severe CKD-aP adult HD patients in the U.S. and Europe in 2021 and 2022, respectively [10]. These approvals were based on the phase 3 KALM-1 and KALM-2 trials [11,12]. Pooled analysis of 851 U.S. patients included in these trials found 51% of patients receiving intravenous (IV) difelikefalin for 12 weeks reported *a* ≥ 3-point reduction in Worst Itching Intensity Numerical Rating Scale (WI-NRS), 55% reported *a* ≥ 15-point reduction in Skindex-10, and 52% reported *a* ≥ 5-point reduction in 5-D itch versus placebo (all *p* ≤ 0.01) [10]. These findings have been supported by a 2024 meta-analysis of 5 RCTs [13].

In RCTs, difelikefalin has demonstrated a significantly higher rate of AEs relative to placebo (RR: 1.26; 95% CI [1.03, 1.55]; *p* = 0.03), with specific differences only in incidence of diarrhea (RR: 3.40; 95% CI [1.54, 7.52]; *p* = 0.003) and dizziness (RR: 2.68; 95% CI [1.23, 5.82], *p* = 0.01) [13]. No differences were observed for serious AEs (*p* = 0.25), AEs leading to treatment discontinuation (*p* = 0.06), or death (*p* = 0.77) compared to placebo [13].

### Objectives

While existing RCTs provide robust evidence for the efficacy and safety of difelikefalin under controlled experimental conditions, the generalizability of results to routine clinical practice remains uncertain. Real-world evidence (RWE) studies are important for various reasons.

RCTs present a highly selective sample of the general population *via* strict inclusion and exclusion criteria, often excluding older patients, those with multiple comorbidities, and those taking other medications [14]. Consequently, patients included in RCTs tend to have fewer comorbidities and a lower risk profile compared to real-world patients [14,15]. Additionally, RCTs tend to underrepresent patients from lower socioeconomic and ethnic minority groups, and these factors are often poorly reported [16,17].

The highly structured monitoring and follow-up of RCTs also leads to higher treatment adherence than clinical practice [15]. Real-world practice involves variable dosing, frequency, adherence, and missed doses that are not reflected to the same extent in clinical trials.

Due to these reasons, a comprehensive systematic review evaluating effectiveness and safety data from all existing RWE studies of difelikefalin is needed to quantify any differences between real-world and clinical trial contexts.

## Methods

### Protocol registration

This systematic review was prospectively registered in the International Prospective Register of Systematic Reviews (PROSPERO) database as CRD420251266218 (available from https://www.crd.york.ac.uk/PROSPERO/view/CRD420251266218). The protocol was conducted in accordance with the Preferred Reporting Items for Systematic Reviews and Meta-Analyses (PRISMA) guidelines (Supplementary Table 1) [18].

### Search strategy

A systematic literature search was conducted across six databases: MEDLINE, Embase, Cochrane Central Register of Controlled Trials (CENTRAL), Cumulative Index to Nursing and Allied Health Literature (CINAHL), Scopus, and Web of Science. A comprehensive search query was developed to identify all relevant English language publications investigating the use of difelikefalin to treat CKD-aP in real-world settings, from database inception to 15 December 2025 (Supplementary Table 2). Forward and backward citation tracking of included studies were performed to identify additional studies. Conference proceedings were included to reduce the risk of publication bias.

### Study selection

Study eligibility was determined using the PICOS framework. The inclusion criteria were as follows:

- Population (P): Adult patients (≥18 years) with CKD-aP.
- Intervention (I): Difelikefalin administered via any dose, frequency, route, or regimen.
- Comparator (C): None required.
- Outcomes (O): One of the following: pruritus severity (WI-NRS ≥3-point response, 5-D Itch Scale), QoL (Skindex-10, EQ-5D, SF-12), sleep disturbance, treatment persistence/discontinuation, predictors of response, or safety/AEs.
- Study design (S): RWE including prospective/retrospective observational cohort studies, case series (*n* ≥ 5), retrospective database analyses, and open-label extensions of RCTs.

Exclusion criteria included RCTs without open-label extension data, pruritus from non-CKD causes, animal/preclinical studies, and single case reports. Full inclusion and exclusion criteria are outlined in Supplementary Table 3.

Title and abstract screening, followed by full-text review, was conducted independently by two reviewers (RS, AG) using Covidence, with conflicts resolved by discussion.

### Data extraction and quality assessment

Data extraction and quality assessment were performed by two independent reviewers (MW, HYC), with conflicts resolved by a third reviewer (RS). The data extraction tool captures study identifiers, study design and setting, population characteristics, intervention details, outcome measures and results, and quality/risk of bias indicators. Supplementary Table 4 presents full details on data points extracted.

Two complementary risk of bias assessment tools were used. ROBINS-I was used for non-randomized interventional and cohort studies [19]. The Joanna Briggs Institute (JBI) Critical Appraisal Checklist was used for case series [20]. Certainty of evidence for primary outcomes (pruritus severity, QoL, sleep disturbance, AEs) was assessed using the GRADE approach [21].

### Data analysis

A quantitative meta-analysis was not possible due to the heterogeneity in design, duration, and outcome measures of included studies. Therefore, findings are tabulated and presented in a narrative synthesis to compare findings across study designs for each reported outcome.

## Results

### Study selection

**Figure 1.**
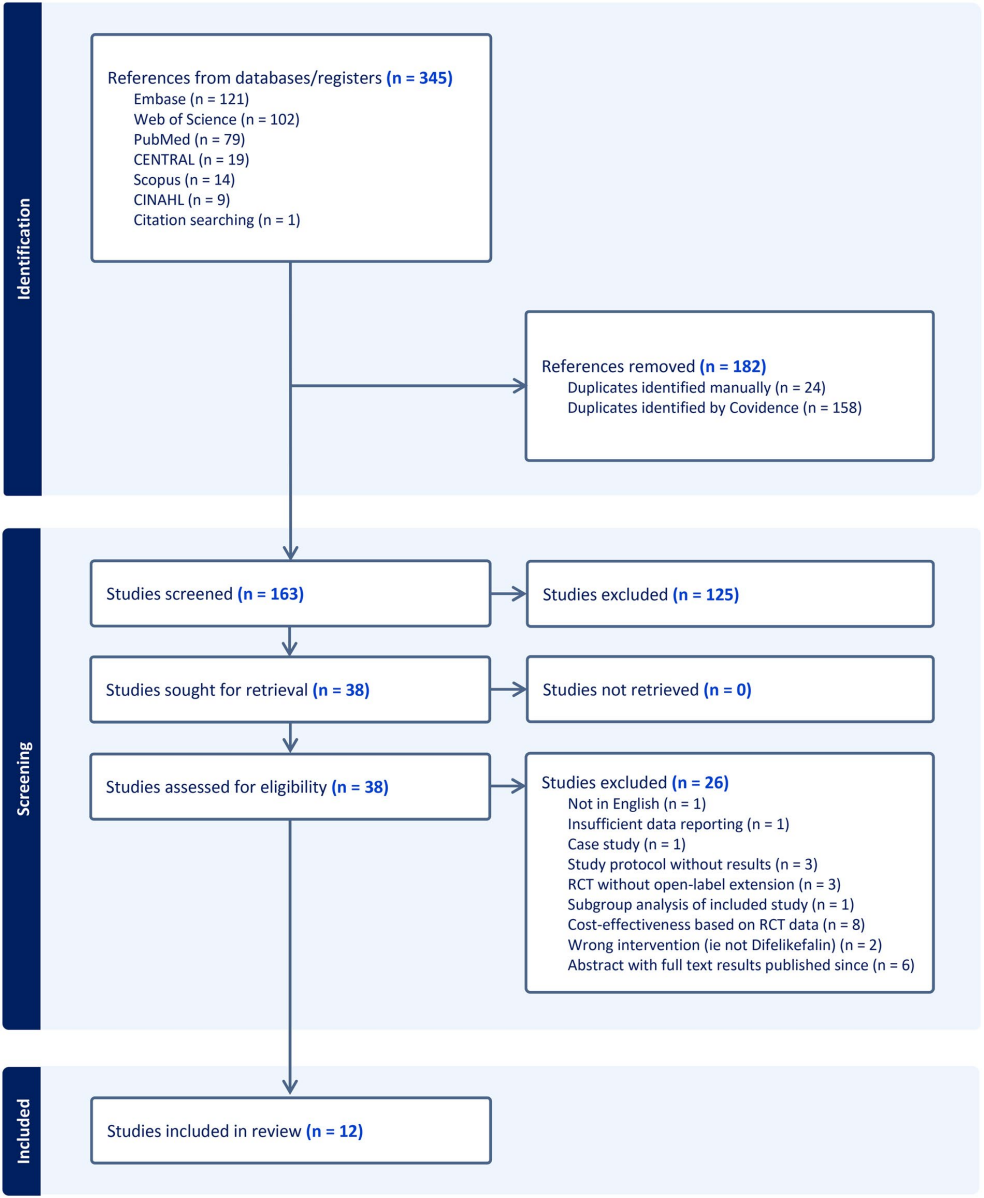
PRISMA flow diagram. Legend: CENTRAL, Cochrane Central Register of Controlled Trials; CINAHL, Cumulative Index to Nursing and Allied Health Literature; RCT, Randomized Controlled Trial.

The systematic search identified 345 studies across six databases. After removal of duplicates, 163 titles and abstracts were screened along with 38 full-text articles, which were assessed for eligibility. Twelve studies met the final inclusion criteria and were included in the narrative synthesis. These included six real-world observational studies [22–27], one large retrospective database analysis [28], three case series [29–31], and two RCTs with open‑label extensions [10,32]. A PRISMA flow diagram documents the screening process (Figure 1). Tables 1 and 2 outline study characteristics and outcomes, respectively.

**Table 1.** Study characteristics.

Study	Country	Study design	N	Mean Age (years)*	Baseline WI-NRS*	Treatment duration*	Dialysis vintage (years)*	Etiology	Other medications	Ethnicity
Aucella (2025) [22]	Italy	Retrospective observational	20	72 ± 11	8.2	12 weeks	4.5 ± 3.7	NR	**Concomitant**: NR total Topical skin lotions: 12 (60%) Antihistamines: 7 (35%) Gabapentin/pregabalin: 6 (30%) **Prior**: 20 (100%) Antihistamines: 19 (95%) Topical skin lotions: 17 (85%) Gabapentin/pregabalin: 8 (40%) Antidepressants: 5 (25%) Cortisonic: 1 (5%)	NR
			(13M, 7F)							
Brasme (2025) [23]	France	Retrospective observational	25	72	8 (IQR 7-8.5)	33 weeks (IQR 26–45) before discontinuation	2.3 (IQR 1.6-10)	Diabetic nephropathy: 40%	**Concomitant**: NR total Emollient: 18 (72.0%) Antihistamines: 12 (48.0%) Pregabalin: 4 (16.0%) Local corticosteroids: 2 (8.0%) Gabapentin: 1 (4.0%) Systemic corticosteroids: 1 (4.0%) **Prior**: NR	NR
			(12M, 13F)	(IQR 68–79)				Vascular disease/nephrosclerosis: 32%		
Di Giovanni (2025) [24]	Italy	Retrospective observational	20	70.8 ± 12.89	8 ± 0.97	14/20 (70%) completed 26 weeks; 12/20 (60%) completed 52 weeks	4.5 (IQR 9.75)	Diabetes: 3 (15%)	NR	NR
			(10M, 10F)					Hypertension: 2 (10%)		
								Glomerulonephritis: 5 (25%)		
								Other/unknown: 10 (50%)		
Ficociello (2025) [28]	USA (Fresenius)	Retrospective database	715	64.7 ± 13	8.5 ± 1.7	Variable (156/715 [22%] had 12-week follow-up)	4.8 ± 4.5	NR	NR	Black: 285 (39.9%) Hispanic: 68 (9.5%)
			(370M, 345F)							
Ficociello (a) Complete regimen group			295	66.1 ± 12.9	8.5 ± 1.8		4.4 ± 4.2	NR	NR	Black: 113 (38.3%) Hispanic: 29 (9.8%)
			(171M, 124F)							
Ficociello (b) Incomplete regimen group			420	63.8 ± 13.1	8.5 ± 1.6		5.2 ± 4.6	NR	NR	Black: 172 (41.0%) Hispanic: 39 (9.3%)
			(199M, 221F)							
Fishbane (2022) [32]	USA, Canada, Czechia, UK, Hungary, Poland, Australia, New Zealand, South Korea, Taiwan	RCT with open-label extension	1306	58.3 ± 12.8	Moderate-severe	Up to 52 weeks	4 (IQR 5.2)	NR	**Concomitant**: NR **Prior**: 517 (39.6%) Diphenhydramine: 321 (24.6%) Hydroxyzine: 131 (10.0%) Hydrocortisone: 30 (2.3%) Cetirizine: 20 (1.5%) Clemastine: 18 (1.4%)	White: 692 (53.0%) Black: 494 (37.8%) Asian: 57 (4.4%) Other: 58 (4.4%)
Kraft (2023) [30]	Germany, Austria, France	Case series	15	73	8 (IQR 7–9)	NR	4 (IQR 2–5)	NR	**Concomitant**: 11 (73.3%) Pregabalin: 5 (33.3%) Mirtazapine: 4 (26.7%) Gabapentin: 3 (20.0%) Lorazepam: 3 (20.0%) Hydroxyzine: 2 (13.3%) Dimetindene: 2 (13.3%) **Prior**: 15 (100%) Pregabalin: 8 (53.3%) Hydroxyzine: 5 (33.3%) Mirtazapine: 4 (26.7%) Gabapentin: 3 (20.0%) Dimetindene: 3 (20.0%) Lorazepam: 3 (20.0%)	NR
			(8M, 7F)	(IQR 57–81)						
Latus (2025) [25]	Europe, Australia	Managed Access Program (prospective)	438	70 ± 13.7	Moderate-severe (≥4 WI-NRS)	Median 16.4 weeks (range 4–148)	NR	NR	NR	NR
Lemy (2025) [27]	Belgium	Open-label single-arm intervention trial	28	NR	NR	52 weeks	NR	NR	NR	NR
Mejia (2024) [29]	Spain	Case series	14	NR	>7	4 weeks	NR	NR	NR	NR
Takahashi (2025) [31]	Japan	Case series	9	70	NR	16 weeks	3.9 (IQR 2.6–5.9)	Diabetes mellitus: 5 (55.6%)	**Concomitant**: 9 (100%) Moisturizers: 9 (100%) Topical steroids: 9 (100%) Other topical drugs: 4 (44.4%) Oral antihistamines: 3 (33.3%) GABA analogues: 2 (22.2%) **Prior**: 9 (100%) Nalfurafine: 9 (100%)	NR
			(5M, 4F)	(IQR 69–71)				Chronic glomerulonephritis: 3 (33.3%)		
								Nephrosclerosis: 1 (11.1%)		
Topf (2022) [10]	USA, Canada, Czechia, UK, Hungary, Poland, Australia, New Zealand, South Korea, Taiwan	RCT with open-label extension	712	Placebo/Difelikefalin: 58.3 ± 13.5, Difelikefalin: 59.1 ± 12.4	Placebo/Difelikefalin: 7.2 ± 1.5, Difelikefalin: 7.2 ± 1.4	Up to 52 weeks	3.5 (IQR 4.8)	Diabetes: 431 (57.2%)	NR	NR
								Hypertension: 260 (34.5%)		
								Glomerulonephritis: 34 (4.5%)		
								Cystic kidney: 29 (3.8%)		
Weiner (2022) [26]	USA, Czechia, Hungary, Poland	Open label single-arm intervention trial	222	58.1 ± 12.8	7.6	12 weeks	NR	Hypertension: 135 patients (60.8%)	**Concomitant**: 71 (32.0%) Diphenhydramine: 49 (22.1%) Hydroxyzine: 14 (6.3%) Gabapentin: 3 (1.4%) **Prior**: 46 (20.7%) Gabapentin: 46 (20.7%)	Black: 110 (49.5%) White: 96 (43.2%) Other: 16 (7.2%)
			(121M, 101F)		(95% CI 7.4, 7.7)			Diabetes: 110 patients (49.5%)		
								Glomerulonephritis: 11 patients (5.0%)		
								Other: 25 patients (11.3%)		

Legends: CI, confidence interval; F, female; IQR, interquartile range; M, male; NR, not reported; RCT, randomized controlled trial; UK, United Kingdom; USA, United States of America; WI NRS, worst itch-numerical rating scale. *reported as mean ± standard deviation, mean (95% confidence interval), or median (interquartile range).

**Table 2.** Primary outcomes of included studies.

Study	WI-NRS responders (decreased by ≥3 points)	WI-NRS	5D-Itch	Skindex-10	Sleep	Quality of life (other)	Any AE	Mortality	Treatment persistence
Aucella (2025) [22]	16/18 (88.9%)	Baseline: 8.2 ± 0.8 Week 4: 4.7 ± 2.9 Week 12: 3.4 ± 2.8 Mean change: *p* < 0.004	Baseline: 18.6 ± 2.6 Week 12: 10.5 ± 3.8 Mean change: −8.1 (43.5% reduction, *p* < 0.003)	NR	Scale: SQ-NRS Baseline: 2.6 ± 1.81 Week 12: 5.8 ± 2.36 Change: *p* < 0.003	Itch-related QoL significant improvement (*p* < 0.003 in SADS)	2 discontinuations (dizziness, discomfort)	NR	18/20 completed 12-week follow-up
Brasme (2025) [23]	N/A	Baseline: 8 (IQR 7-8.5) at discontinuation of difelikefalin Relapse: 7 (IQR 5.25–8) One month after resumption: 4.5 (IQR 2.25–5.5)	NR	Baseline: 9.2 (IQR 2-16.75) at discontinuation of difelikefalin Relapse: 22 (IQR 13.25–31.25) One month after resumption: 12 (IQR 6.25–24.25)	NR	NR	NR	1 patient died between difelikefalin discontinuation and 3-month follow-up; cause of death not specified	Median 7.6 months before discontinuation of difelikefalin; 52% required reinitiation within 3 months post-discontinuation
DiGiovanni (2025) [24]	NR	Baseline: 8 ± 0.97 Week 52: 3.92 ± 3.32 Mean change: *p* < 0.001	NR	NR	Scale: PSDS Baseline: 2.9 ± 1.07 Week 52: 1.17 ± 1.34 Change: *p* = 0.002	Scale: SF-12 physical Baseline: 31.51 ± 7.92 Week 52: 31.9 ± 10.15 Change: *p* > 0.05 Scale: SF-12 mental Baseline: 41.73 ± 11.43 Week 52: 48.36 ± 12.42 Change: *p* > 0.05	3 discontinuations (2 diarrhea, 1 mild cognitive impairment)	3/20 (15%) unrelated to difelikefalin	14/20 (70%) completed 6 months; 12/20 (60%) completed 12 months
Ficociello (2025) [28]	72/156 (46%)	Baseline: 9 (IQR 7.5–10) Week 12: 6 (IQR 2–8)	NR	NR	NR	NR	Multiple events recorded 223 Significant AEs: Dizziness: (*P* = 0.0027) Hyperkalaemia: (*P* = 0.0001) Other AEs: nausea, diarrhea, headache, vomiting	NR	Highly variable; 156/715 (22%) had 12-week follow-up
Ficociello (a) Complete regimen group (CRG)	47/84 (56%)	Baseline: 9 (IQR 7–10) Week 12: 5 (IQR 2–7) Mean Change: *p* = 0.008	NR	NR	NR	NR	NR	NR	NR
Ficociello (b) Incomplete regimen group (IRG)	25/72 (35%)	Baseline: 9 (IQR 8–10) Week 4: NR Week 12: 8 (IQR 5–10) CRG group significantly higher WI-NRS response rate compared to IRG group (*p* = 0.008)	NR	NR	NR	NR	NR	NR	NR
Fishbane (2022) [32]	NR	NR	NR	NR	NR	NR	AEs: diarrhea, dizziness, nausea, hyperkalaemia, somnolence, altered mental state, headache and gait disturbances	4.3% (unrelated to difelikefalin)	NR
Kraft (2023) [30]	13/15 (87%)	Baseline: 8 (IQR 7–9) Endpoint: 3 (IQR 1.75-5)	NR	NR	NR	NR	Dizziness (2), Diarrhea (1), Headache (2), Fatigue (1), Deaths unrelated to difelikefalin (2)	2 deaths (unrelated to difelikefalin; lung cancer, sepsis)	Variable; at least 10 continued some follow-up
Latus (2025) [25]	NR	NR	NR	NR	NR	NR	AEs in 167/458 (38.1%)	63 fatal SAE (unrelated to difelikefalin)	Median 115 days (range 30–1035 days)
Lemy (2025) [27]	NR	Baseline: NR Mean change: significant decrease from baseline at 1 year (*p* = 0.006)	Baseline: NR Change: significant decrease from baseline at 1 year (*p* = 0.019)	Change: significant decrease from baseline at 1 year (*p* = 0.023)	NR	No significant change in anxiety/depression scores	NR	NR	8/28 completed 1 year of treatment
Mejia (2024) [29]	11/14 (78.5%) at 2 weeks 13/14 (92.9%) at 4 weeks	NR	NR	NR	NR	SADS C (severe deterioration of QoL) -> SADS A (mild deterioration)	AE in 5/14 (36%); diarrhea, nausea, hypotension, dizziness	NR	11/14 continuing at endpoint (4 weeks)
Takahashi (2025) [31]	NR	Scale: Daytime Shiratori itch scale Baseline: 3 very mild, 2 mild, 3 moderate, 1 severe Week 16: 1 no symptoms, 6 very mild, 2 mild Change: *p* < 0.001	NR	NR	Scale: Nighttime Shiratori itch scale Baseline: 3 mild, 5 moderate, 1 severe Week 16: 2 no symptoms, 5 very mild, 2 mild Change: *p* < 0.001	Insomnia 4 -> 0 Treatment satisfaction Baseline: 4 satisfied, 3 neither satisfied nor dissatisfied, 2 dissatisfied Week 16: 1 very satisfied, 8 satisfied	0 (0%)	NR	9/9 completed 16-week follow-up
Topf (2022) [10] – Difelikefalin	NR	NR	Baseline: NR Change: increased from 172/331 (52%) to 70/92 (76.1%) at 52 weeks	NR	NR	NR	NR	NR	NR
Topf (2022) [10] – Placebo/Difelikefalin	NR	NR	Baseline: NR Change: increased from 153/364 (42%) to 76/97 (78.4%) at 52 weeks	NR	NR	NR	NR	NR	NR
Weiner (2022) [26]	144/194 (74%)	Baseline: 7.6 (95% CI 7.4, 7.7) Week 12: 3 (95% CI 2.7, 3.3) Mean Change: −4.5 (95% CI −4.9, −4.2), *p* < 0.001	Baseline: 17.1 (95% CI 6.3, 6.9) Week 12: 10.1 (95% CI 9.6, 10.5) Mean change: −7.1 (95% CI, −7.7 to −6.5), *p* < 0.001, 69.8% response rate	Baseline: 32.9 (95% CI 31, 34.8) Week 12: 12.3 (95% CI 10.5, 14.1) Mean change: −21.0 (95% CI −23.2, −18.7), *p* < 0.001, 63.0% response rate	Scale: SQ-NRS Baseline: 6.6 (95% CI 6.3, 6.9) Week 12: 2.4 (95% CI 2.1, 2.7) Mean change: −4.3 (95% CI −4.6, −3.9), *p* < 0.001	Scale: EQ-5D VAS Baseline 68.4 (95% CI 65.9, 70.9) Week 12: 70.7 (95% CI 67.7, 73.6) Mean change: 2.3 (95% CI 0.0, 5.5); *p* = 0.046	Any AEs occurred in 143 (64.4%) patients Treatment-related AEs occurred in 16 (7.2%) patients	NR	197/222 (88.7%) completed 12 weeks

Legends: AE, adverse event; CI, confidence interval; CRG, complete response group; EQ-5D VAS, euroqol-5 dimensions visual analogue score; IRG, incomplete response group; NR, not reported; PSDS, pruritus-related sleep disturbance scale; QoL, quality of life; SADS, self-assessed disease severity scale; SAE, serious adverse event; SF-12, short-form 12-item health survey; SQ-NRS, sleep quality numerical rating scale; WI-NRS, worst itch numerical rating scale.

Sample sizes ranged from 9 to 1306 of patients with CKD-aP on HD with a mean vintage between 2.3 and 5.2 years. The mean ages of patients ranged from 58 to 73 years, with the percentage of male patients ranging from 47% to 65%. Ethnicity was only reported by three studies, ranging from 43% to 53% white participants [26,28,32]. CKD etiology of patients was reported in five studies, predominated by diabetic and hypertensive nephropathy [10,23,24,26,31]. Average baseline dialysis adequacy, as measured by *Kt*/*V*, ranged from 1.33 to 1.86 across five studies [22–24,30,31].

All studies investigated intravenous difelikefalin in adult HD patients with moderate-severe CKD-aP. Takahashi et al. [31] was the only study to deviate from the 3-weekly 0.5 mcg/kg post-dialysis regimen, instead using 17.5 mcg when the dry weight was <45 kg and 25 mcg when the dry weight was 45–65 kg.

The three cohort and case series that declared prior anti-pruritic agent use were reported in all patients [22,30,31], which was maintained throughout the follow-up period. Open-label interventional studies reported lower rates of prior anti-pruritic use between 21% and 40% [26,32]. Five studies reported use of concomitant anti-pruritic medication usage, including emollients, antihistamines, gabapentin/pregabalin, topical steroids, and opioid medications [22,23,26,30,31]. Studies were generally at a high risk of bias due to limited control for confounding factors, with only one study – Weiner et al. [26] – rated as moderate risk of bias.

### Pruritus severity

The commonest tool to assess pruritus severity was WI-NRS in seven studies [22–24,26,28–30]. This tool asks patients to rate the severity of their worst itch in the past 24 h on a scale from 0 to 10. Rates of a clinically significant reduction in severity, defined as *a* ≥ 3 point reduction in WI-NRS score [33], ranged from 35% to 93%.

Large, multicenter datasets displayed positive results. Weiner et al. [26], a 12-week open-label trial of 222 participants, reported a mean reduction in WI-NRS score of −4.5 (95% CI −4.9, −4.2; *p* < 0.001) and a 74% response rate. Subgroup analysis of the Fresenius database reported patients receiving the complete difelikefalin regimen demonstrated a significantly greater WI-NRS response rate compared to the incomplete regimen group (56% vs 35%; *p* = 0.008), indicating that continuous treatment adherence is important for difelikefalin efficacy in CKD-aP [28].

Short-term observational studies up to 12 weeks consistently showed significant and duration-dependent reductions in pruritus severity with difelikefalin use. In Aucella et al. [22], a retrospective analysis of 20 patients in Italy, the baseline WI-NRS score of 8.2 ± 0.8 reduced to 4.7 ± 2.9 at 4 weeks and 3.4 ± 2.8 after 12 weeks (*p* < 0.004), with 88.9% of patients achieving *a* ≥ 3‑point WI‑NRS reduction. Kraft et al. [30], a case series of 15 patients, reported similar reductions in WI-NRS, with a median time to response of 2 weeks (IQR 2, 5.5). Longer-term follow-up studies displayed sustained efficacy. Lemy et al. [27], a single-arm interventional study of 28 patients, and Di Giovanni et al. [24], a retrospective observational study of 20 patients, both reported significant reductions in WI-NRS score at 12 months.

Four studies used the 5-D itch scale, which assesses pruritus in the past 2 weeks over 5 domains: degree, duration, direction, disability, and distribution [34]. All studies showed significant reductions in the 5-D itch score [10,22,26,27]. Weiner et al. [26] reported a mean change of −7.1 (95% CI −7.7, −6.5); *p* < 0.001; 69.8% response rate at 12 weeks. Additionally, pooled analysis of the 1-year open label extensions of the KALM studies demonstrated increases in the number of participants achieving a clinically significant reduction in 5-D itch score (≥5 points) [10].

### Sleep disturbance and QoL

Sleep quality improved significantly in all four studies that reported it: at 12 weeks in Weiner’s single-arm interventional and Aucella’s retrospective observational studies [22,26], 16 weeks in Takahashi’s case series [31], and 1 year in Di Giovanni’s retrospective observational study [24]. These studies support the significant improvements in sleep quality reported by one RCT [35]; however, results should be further validated in additional higher-powered studies.

All studies reporting pruritus-specific QoL outcomes showed significant improvements. Weiner et al. [26] reported a significant reduction in pruritus-related QoL using Skindex-10 (mean change −21.0 [95% CI −23.2, −18.7; *p* < 0.001]; 63.0% response rate). Additionally, Aucella et al. [22] reported a significant improvement in pruritus-related QoL using the Self-Assessed Disease Severity scale (*p* < 0.003).

Regarding generic QoL tools, the results were mixed. The highest quality real-world study, Weiner et al. [26] reported a significant improvement in EQ-5D VAS (mean change 2.3 [95%CI 0.0, 5.5; *p* = 0.046]). Di Giovanni et al. [24], a retrospective cohort study of 20 patients, also reported QoL using SF-12. While no significant improvement in the SF-12 physical component summary was reported between baseline (31.51 ± 7.92) and 12 months (31.9 ± 10.15; *p* > 0.05), a trend toward improvement was seen in the mental component summary (41.73 ± 11.43 at baseline, 48.36 ± 12.42 at 12 months; *p* < 0.05).

These real-world studies indicate that difelikefalin’s anti-pruritic effect may lead to improvements in pruritus-specific QoL and sleep scores, but the effect on overall QoL is unclear.

### Treatment persistence, discontinuation, and recurrence

Treatment persistence was variable across the studies, often lower in longer-term studies. In Weiner et al. [26], the highest quality included study, 88.7% (197/222) completed the 12‑week open‑label period [26]. Similar rates were reported in a short-term case series and observational study. In Takahashi et al. [31], all nine patients completed 16 weeks of treatment. In Aucella et al. [22], 18/20 patients (90%) completed the 12-week treatment course, with the two discontinuations related to dizziness and non‑specific discomfort.

In longer-term studies like Di Giovanni et al. [24], 14/20 patients (70%) remained on difelikefalin at 6 months and 12/20 (60%) at 12 months. Higher rates of discontinuation were observed by Lemy et al. [27], with only 8/28 (28.6%) patients completing 1 year of treatment, with withdrawals attributed to a lack of efficacy or mild AEs.

Brasme et al. [23] (*n* = 25) intentionally discontinued difelikefalin in patients who had achieved sustained pruritus control to examine recurrence. Patients had received a median of 7.6 months of difelikefalin before stopping and had median WI‑NRS scores of 2 (IQR 0–3) and Skindex-10 scores of 9.2 (IQR 2–16.75) at discontinuation. Within 3 months of withdrawal, 52% (13/25) required reinitiation due to recurrence of CKD‑aP, with median WI‑NRS score rising to 7 (IQR 5.25–8) and Skindex-10 score increasing to 22 (IQR 13–31) at relapse. Mean event‑free survival after discontinuation was 69 ± 6 days. One month after re-initiating difelikefalin, the median WI‑NRS and Skindex-10 scores decreased to 4.5 (IQR 2.25–5.5) and 12 (IQR 6.25–24.25).

These findings suggest that long-term use of difelikefalin may be associated with reduced treatment persistence for several reasons. Around half of individuals experienced worsening pruritus within three months of withdrawal, with symptomatic improvement observed upon treatment reinitiation.

### Safety and tolerability

Safety and tolerability were reported across nine studies but varied substantially in completeness, attribution to difelikefalin, and follow-up duration. In studies with comprehensive reporting, overall AEs were identified in 38.1–64.4% of patients.

In Weiner et al. [26], AEs occurred in 143 patients (64.4%), with 16 (7.2%) related to difelikefalin. The most frequently reported AEs include diarrhea (5%), nausea (4.5%), hyperkalaemia (4.1%), headache (3.6%), hypertension (3.6%), nasopharyngitis (3.6%), and dizziness (3.2%). 14 (6.3%) of AEs led to treatment discontinuation.

Latus et al. [25] reported the safety findings of a European and Australian managed access program. 38.1% (167/458) patients experienced a total of 458 AEs. Of these, 246 were classed as serious AEs, which were experienced by 87 patients. 25 (10.2%) of serious AEs were considered to have a possible relation to difelikefalin, most commonly hyperkalemia (16%), a confusional state (12%), and nausea (12%). 86.4% of treatment-related serious AEs were resolved by the time of data collection. There were 63 fatal events with 43 deaths, with none linked to difelikefalin usage; the most involved systems were cardiac (28.6%), general disorders and administration site conditions (23.8%), and infections (22.2%).

Ficociello et al. [28] reported AEs in patients using the U.S. Fresenius database. A statistically significant increase in dizziness (0.09–0.2%; *p* = 0.0027) and hyperkalemia (2.0% to 2.6%; *p* = 0.0001) was found from before and after difelikefalin administration. Pooled open-label extension data from the KALM studies identified diarrhea, dizziness, nausea, somnolence, and gait disturbance as the most frequent AEs, with no new safety signals after 52 weeks of treatment [32].

Overall, the frequency and pattern of AEs appear to be similar to those previously identified in RCTs. However, their clinical significance in CKD-aP patients, often with significant frailty and co-morbidity, remains to be determined.

### Quality assessment and GRADE evaluation

The quality of included studies was generally poor due to their observational, real-world nature – thus substantially weakening the certainty of findings. Seven of eight observational studies were found to be at severe risk of bias using the ROBINS-I tool, with Weiner et al. [26] rated as moderate risk of bias. The predominant causes of the significant risk of bias were confounding factors (no adjustment for baseline pruritus severity, concomitant medications, or dialysis adequacy), deviation from intended interventions (variable adherence, incomplete regimens), and missing outcome data (dropout rates 10–71% in longer-term studies) (Table 3).

**Table 3.** Risk of bias in observational studies using ROBINS-I.

Study	Study design	1 Bias due to confounding	2 Bias in classification of interventions	3 Bias in selection of participants into the study	4 Bias due to deviations from intended interventions	5 Bias due to missing data	6 Bias in measurement of outcomes	7 Bias in selection of the reported result	Overall
Aucella (2025) [22]	Retrospective observational	Serious	Low	Low	Low	Serious	NA	Low	Serious
Brasme (2025) [23]	Retrospective observational	Serious	Low	Low	Low	Low	NA	Low	Serious
DiGiovanni (2025) [24]	Retrospective observational	Serious	Low	Low	Serious	Serious	NA	Low	Serious
Ficociello (2025) [28]	Retrospective database	Serious	Low	Low	Serious	Serious	NA	Low	Serious
Fishbane (2022) [32]	RCT with open-label extension	Serious	Low	Low	Serious	Serious	NA	Low	Serious
Latus (2025) [25]	Managed access program (prospective)	Serious	Low	Low	Serious	Serious	NA	Low	Serious
Lemy (2025) [27]	Open-label single-arm intervention trial	ABSTRACT							
Topf (2022) [10]	RCT with open-label extension	Serious	Low	Low	Serious	Serious	NA	Low	Serious
Weiner (2022) [26]	Open-label single-arm intervention trial	Moderate	Low	Low	Low	Low	NA	Low	Moderate

Legend: NA, not applicable; RCT, randomized controlled trial.

Two case series were rated as eight and nine out of 10 using the JBI checklist for case series [30,31]. Points were lost due to the lack of statistical analysis in Kraft et al. [30] and the unclear reporting of whether patients were included consecutively in both studies [30,31]. Two studies were not assessed for risk of bias, as they are conference abstracts with limited reporting of their methods (Tables 3 and 4) [27,29].

**Table 4.** Risk of bias in case series using Joanna Brigg’s Institute critical appraisal checklist.

Study	Country	Study type	1 Clear inclusion criteria	2 Condition measurement	3 Valid methods to identify condition	4 Consecutive inclusion of participants	5 Complete inclusion of participants	6 Clear demographic reporting	7 Clear clinical information reporting	8 Clear outcome reporting	9 Clear reporting site demographic information	10 Appropriate statistical analysis	Score out of 10
Kraft (2023) [30]	Germany, Austria, France	Case series	Yes	Yes	Yes	Unclear	Yes	Yes	Yes	Yes	Yes	Not applicable	8
Mejia (2024) [29]	Spain	Case series	ABSTRACT										
Takahashi (2025) [31]	Japan	Case series	Yes	Yes	Yes	Unclear	Yes	Yes	Yes	Yes	Yes	Yes	9

The certainty of evidence for each primary outcome was assessed using the GRADE approach: pruritus severity, QoL, and sleep disturbance, AEs. As the included studies were predominantly non-randomized and observational, the initial certainty was rated low for all outcomes. Further downgrading was applied to all outcomes due to risk of bias, inconsistency, and imprecision. No upgrading factors were identified; thus, all outcomes were rated as ‘very low’ certainty.

## Discussion

This systematic review identified twelve real-world studies evaluating the use of intravenous difelikefalin for CKD-aP in HD patients. Across included studies, a wide range of 35–93% of patients achieved a clinically significant ≥3-point reduction in WI-NRS, reflecting the methodological heterogeneity, lack of control for confounding factors such as baseline WI-NRS score, and high RoB of included studies. The real-world WI-NRS response rate of 35–93% encompasses the 51–72% WI-NRS responders in four RCTs [10,35–37]. Similarly, the mean reductions in 5-D itch and Skindex-10 scores in real-world studies [22,26] generally surpassed those reported in RCTs at 12 weeks [10,35,36]. All four real-world studies that measured sleep quality reported significant improvements [22,24,26,31], supporting RCT findings [35].

Comparisons between real-world studies and previous RCTs are possible but should be interpreted with caution. Selection bias, attrition bias, placebo effects, and other sources of heterogeneity and bias lead to a very low certainty of evidence for observational studies, thus preventing the establishment of equivalence or superiority compared to RCTs.

While improvements in pruritus-specific QoL and sleep quality tools were consistent, generic QoL tools showed variability [24]. This dissociation is clinically plausible: HD patients carry a substantial physical symptom burden from uremia, fluid overload, and comorbidities that difelikefalin does not address [38–40]. It suggests that studies relying solely on pruritus-specific tools may overstate holistic benefit, and future studies should incorporate both disease-specific and generic instruments to allow more nuanced interpretation of patient benefit.

Real-world safety results up to 12 months in the open-label extension of the KALM trials showed no new safety signals. However, the heterogeneous reporting of safety outcomes and difficulty attributing a specific cause to AEs limits the strength of conclusions. Treatment persistence was generally encouraging in studies up to 16 weeks but was more variable in longer-term studies, with mild AEs contributing to 12/20 (60%) of withdrawals at 12 months in Lemy et al. [27].

Although most AEs were deemed mild to moderate, these symptoms can be clinically significant in HD who tend to be old, frail, and with multiple co-morbidities [40,41]. For example, dizziness and gastrointestinal side effects of opioids may increase the risk of falls and impair fluid balance, potentially leading to poorer outcomes [42,43], as is documented with pregabalin/gabapentin use in CKD-aP [44]. Proactive monitoring, early identification of at-risk patients, and structured patient education on managing side effects are therefore essential for safe real-world deployment. Additionally, an individualized discussion of risks vs benefits should be undertaken prior to the initiation of difelikefalin for CKD-aP for each patient, including the risks of untreated CKD-aP such as increased depression, hospitalization, and mortality [45].

There was substantial variation in use of prior and concomitant medications (Table 1). Weiner et al. [26] reported 69 (31%) patients used concomitant opioids. This was not associated with greater reductions in pruritus severity compared to difelikefalin alone but did show a greater incidence of AEs [46]. This may suggest against the use of concomitant opioids in patients receiving difelikefalin for CKD-aP. Prospective studies investigating optimal concomitant medication strategies alongside difelikefalin initiation are warranted.

### Limitations

There are multiple limitations to consider in the context of the available data. First, the twelve included studies represent a relatively small sample size with a generally high risk of bias. Responder, selective survival, and publication biases are inherent to real-world studies and may explain why real-world efficacy appears to sometimes numerically exceed that reported by RCTs.

Second, there was generally poor reporting and statistical control for potentially important confounding factors. Only three studies reported the ethnicity of participants [26,28,32], and five studies reported each of the following: prior anti-itch medication usage [22,26,30–32], concomitant anti-itch medication during the study period [22,23,26,30,31], primary renal disease etiology [10,23,24,26,31], and dialysis treatment adequacy (*Kt*/*V*) [22–24,30,31] (Table 1). These factors may contribute to the heterogeneity in results of included studies, as seen in the range of WI-NRS response rates from 35% to 93%; however, only one study conducted a subgroup analysis to assess this [26].

There are also limitations related to outcome measurement in studies. The heterogeneous use of tools for assessment of pruritus severity and QoL hinders the feasibility of a meta-analysis to comprehensively synthesize results. WI-NRS, the predominant efficacy measurement, relies exclusively on patients recalling their worst itch severity in the preceding 24 h. Therefore, it is susceptible to recall bias and fails to holistically represent the various aspects of life that CKD-aP may affect [47,48]. This underscores the need for consensus on a minimum outcome dataset for CKD-aP trials, analogous to Core Outcome Measures in Effectiveness Trials (COMET) initiatives in other chronic diseases [49,50].

### Recommendations for future research

All included studies examined intravenous difelikefalin in adult HD patients. Peritoneal dialysis patients experience similar pruritus rates as HD patients (∼55%) [2], and approximately 24% of non-dialysis CKD stage 4–5 patients experience moderate-to-severe pruritus [51]. Kidney transplant recipients with persistent post-transplant pruritus represent a further under-studied group [52]. Future prospective studies should specifically recruit these populations.

A phase 2 RCT demonstrated significant reductions in pruritus severity with once-daily oral difelikefalin in CKD patients, with a broadly comparable safety profile [37]. Oral administration removes the requirement for hospital attendance, making it a more viable formulation for peritoneal dialysis and non-dialysis patients. RWE for oral difelikefalin represents an important evidence gap, particularly as oral formulations generally achieve higher adherence than parenteral regimens in chronic disease management [53].

No included study systematically identified baseline characteristics associated with treatment response. Understanding which patients are most and least likely to benefit from difelikefalin is essential for targeted prescribing in a resource-constrained healthcare environment. Future studies should be powered to conduct subgroup analyses and ideally incorporate biological samples to explore mechanistic predictors of response.

No studies have directly compared difelikefalin to active alternatives such as nalbuphine, nalfurafine, or optimized gabapentinoid regimens. Pragmatic randomized or quasi-experimental comparative effectiveness designs would address this gap and directly inform treatment sequencing decisions.

Difelikefalin is a high-cost licensed agent, but its cost-effectiveness in routine clinical practice has not been evaluated in any real-world study. The potential cost-offset from reduced hospitalization, improved dialysis compliance, and reduced emergency attendance secondary to better pruritus control should be quantified alongside AE-associated costs to enable robust ICER and QALY calculations and inform the development of clinical guidelines.

The heterogeneity of outcome tools across included studies prevented meta-analysis. We propose that future studies should, at minimum, report WI-NRS, 5-D itch, Skindex-10, a sleep quality measure, a kidney disease-specific QoL tool (KDQOL-36), and a validated overall QoL instrument such as EQ-5D.

Future studies should prioritize recruitment of under-represented patients from ethnic minority and lower socioeconomic groups, with mandatory reporting of race, ethnicity, and socioeconomic indicators. Studies from low- and middle-income countries are particularly needed to evaluate the external validity of study findings. Collaborative international registry designs could help address these gaps.

## Conclusion

This systematic review represents the first of its kind investigating the efficacy and safety of difelikefalin in real-world settings. Included studies were generally at high risk of bias with variable results; however, these tentatively support the findings of previous RCTs, with efficacy and safety maintained in studies reporting up to 12 months of follow-up. Although findings regarding overall QoL improvements were inconsistent, significant improvements in pruritus-specific QoL and sleep quality were identified in all studies that reported it, albeit with significant methodological limitations. Future studies should prospectively follow-up patients with standardized outcome measures and should be conducted in more diverse clinical contexts.

## Supplementary Material

Difelikefalin SR Supplementary Tables.docx

## Data Availability

All data relevant to this study are included in the article and its supplementary material. Further enquiries can be directed to the corresponding author.
