# Supplementary material for: Real-world effectiveness and safety of difelikefalin for chronic kidney disease-associated pruritus: a systematic review
Source: Ren Fail. 2026 Aug 11;48(1):2711185. doi: 10.1080/0886022X.2026.2711185 (PMC13463438; doi:10.1080/0886022X.2026.2711185)
Supplement: Supplementary file 1 [file IRNF_A_2711185_SM2821.docx]

**Real-World Effectiveness and Safety of Difelikefalin for Chronic Kidney Disease-Associated Pruritus: A Systematic Review**

**Supplementary Tables**

Supplementary Table 1: PRISMA checklist.

Supplementary Table 2: Full search queries for each database.

Supplementary Table 3: Study inclusion and exclusion criteria.

Supplementary Table 4: Outcomes for data extraction.

**Supplementary Table 1: PRISMA checklist.**

| **Section and Topic** | **Item #** | **Checklist item** | **Location where item is reported** |
| --- | --- | --- | --- |
| **TITLE** | | |  |
| Title | 1 | Identify the report as a systematic review. | Page 1 |
| **ABSTRACT** | | |  |
| Abstract | 2 | See the PRISMA 2020 for Abstracts checklist. | Pages 2-3 |
| **INTRODUCTION** | | |  |
| Rationale | 3 | Describe the rationale for the review in the context of existing knowledge. | Pages 4-5 |
| Objectives | 4 | Provide an explicit statement of the objective(s) or question(s) the review addresses. | Page 6 |
| **METHODS** | | |  |
| Eligibility criteria | 5 | Specify the inclusion and exclusion criteria for the review and how studies were grouped for the syntheses. | Page 7-8 |
| Information sources | 6 | Specify all databases, registers, websites, organisations, reference lists and other sources searched or consulted to identify studies. Specify the date when each source was last searched or consulted. | Page 7 |
| Search strategy | 7 | Present the full search strategies for all databases, registers and websites, including any filters and limits used. | Supplementary Table 2 |
| Selection process | 8 | Specify the methods used to decide whether a study met the inclusion criteria of the review, including how many reviewers screened each record and each report retrieved, whether they worked independently, and if applicable, details of automation tools used in the process. | Page 8 |
| Data collection process | 9 | Specify the methods used to collect data from reports, including how many reviewers collected data from each report, whether they worked independently, any processes for obtaining or confirming data from study investigators, and if applicable, details of automation tools used in the process. | Page 8 |
| Data items | 10a | List and define all outcomes for which data were sought. Specify whether all results that were compatible with each outcome domain in each study were sought (e.g. for all measures, time points, analyses), and if not, the methods used to decide which results to collect. | Supplementary Table 4 |
|  | 10b | List and define all other variables for which data were sought (e.g. participant and intervention characteristics, funding sources). Describe any assumptions made about any missing or unclear information. | Supplementary Table 4 |
| Study risk of bias assessment | 11 | Specify the methods used to assess risk of bias in the included studies, including details of the tool(s) used, how many reviewers assessed each study and whether they worked independently, and if applicable, details of automation tools used in the process. | Page 8 |
| Effect measures | 12 | Specify for each outcome the effect measure(s) (e.g. risk ratio, mean difference) used in the synthesis or presentation of results. | Table 2 |
| Synthesis methods | 13a | Describe the processes used to decide which studies were eligible for each synthesis (e.g. tabulating the study intervention characteristics and comparing against the planned groups for each synthesis (item #5)). | Page 9 |
|  | 13b | Describe any methods required to prepare the data for presentation or synthesis, such as handling of missing summary statistics, or data conversions. | NA |
|  | 13c | Describe any methods used to tabulate or visually display results of individual studies and syntheses. | Tables 1-2 |
|  | 13d | Describe any methods used to synthesize results and provide a rationale for the choice(s). If meta-analysis was performed, describe the model(s), method(s) to identify the presence and extent of statistical heterogeneity, and software package(s) used. | Page 9 |
|  | 13e | Describe any methods used to explore possible causes of heterogeneity among study results (e.g. subgroup analysis, meta-regression). | NA |
|  | 13f | Describe any sensitivity analyses conducted to assess robustness of the synthesized results. | NA |
| Reporting bias assessment | 14 | Describe any methods used to assess risk of bias due to missing results in a synthesis (arising from reporting biases). | NA |
| Certainty assessment | 15 | Describe any methods used to assess certainty (or confidence) in the body of evidence for an outcome. | Page 8 |
| **RESULTS** | | |  |
| Study selection | 16a | Describe the results of the search and selection process, from the number of records identified in the search to the number of studies included in the review, ideally using a flow diagram. | Page 9 |
|  | 16b | Cite studies that might appear to meet the inclusion criteria, but which were excluded, and explain why they were excluded. | Page 9 |
| Study characteristics | 17 | Cite each included study and present its characteristics. | Table 1 |
| Risk of bias in studies | 18 | Present assessments of risk of bias for each included study. | Tables 3-4 |
| Results of individual studies | 19 | For all outcomes, present, for each study: (a) summary statistics for each group (where appropriate) and (b) an effect estimate and its precision (e.g. confidence/credible interval), ideally using structured tables or plots. | Table 2 |
| Results of syntheses | 20a | For each synthesis, briefly summarise the characteristics and risk of bias among contributing studies. | Page 10; Pages 15-16 |
|  | 20b | Present results of all statistical syntheses conducted. If meta-analysis was done, present for each the summary estimate and its precision (e.g. confidence/credible interval) and measures of statistical heterogeneity. If comparing groups, describe the direction of the effect. | NA |
|  | 20c | Present results of all investigations of possible causes of heterogeneity among study results. | NA |
|  | 20d | Present results of all sensitivity analyses conducted to assess the robustness of the synthesized results. | NA |
| Reporting biases | 21 | Present assessments of risk of bias due to missing results (arising from reporting biases) for each synthesis assessed. | NA |
| Certainty of evidence | 22 | Present assessments of certainty (or confidence) in the body of evidence for each outcome assessed. | Page 16 |
| **DISCUSSION** | | |  |
| Discussion | 23a | Provide a general interpretation of the results in the context of other evidence. | Pages 16-18 |
|  | 23b | Discuss any limitations of the evidence included in the review. | Pages 18-19 |
|  | 23c | Discuss any limitations of the review processes used. | Pages 18-19 |
|  | 23d | Discuss implications of the results for practice, policy, and future research. | Pages 20-21 |
| **OTHER INFORMATION** | | |  |
| Registration and protocol | 24a | Provide registration information for the review, including register name and registration number, or state that the review was not registered. | Page 7 |
|  | 24b | Indicate where the review protocol can be accessed, or state that a protocol was not prepared. | Page 7 |
|  | 24c | Describe and explain any amendments to information provided at registration or in the protocol. | NA |
| Support | 25 | Describe sources of financial or non-financial support for the review, and the role of the funders or sponsors in the review. | Page 23 |
| Competing interests | 26 | Declare any competing interests of review authors. | Page 23 |
| Availability of data, code and other materials | 27 | Report which of the following are publicly available and where they can be found: template data collection forms; data extracted from included studies; data used for all analyses; analytic code; any other materials used in the review. | Page 23 |

**Supplementary Table 2: Full search queries for each database.**

| **Database** | **Search terms** |
| --- | --- |
| PubMed (MEDLINE) | ( difelikefalin[Title/Abstract] OR Korsuva[Title/Abstract] OR Kapruvia[Title/Abstract] OR  "CR 845"[Title/Abstract] OR CR-845[Title/Abstract] OR CR845[Title/Abstract] OR "kappa  opioid receptor agonist"[Title/Abstract] ) AND ( "real-world"[Title/Abstract] OR "real  world"[Title/Abstract] OR "real-world evidence"[Title/Abstract] OR "real-world  data"[Title/Abstract] OR "real-world use"[Title/Abstract] OR RWE[Title/Abstract] OR  observational[Title/Abstract] OR observational study[Title/Abstract] OR observational  studies[Title/Abstract] OR "real-life"[Title/Abstract] OR "real life"[Title/Abstract] OR  "clinical practice"[Title/Abstract] OR "clinical experience"[Title/Abstract] OR "routine  clinical practice"[Title/Abstract] OR effectiveness[Title/Abstract] OR effectiveness  study[Title/Abstract] OR registry[Title/Abstract] OR registries[Title/Abstract] OR "disease  registry"[Title/Abstract] OR database[Title/Abstract] OR databases[Title/Abstract] OR  "electronic health record"[Title/Abstract] OR "electronic health records"[Title/Abstract]  OR EHR[Title/Abstract] OR claims[Title/Abstract] OR "claims data"[Title/Abstract] OR  "post-marketing"[Title/Abstract] OR "post marketing"[Title/Abstract] OR "post-  market"[Title/Abstract] OR postapproval[Title/Abstract] OR post-approval[Title/Abstract]  OR "actual use"[Title/Abstract] OR pragmatic[Title/Abstract] OR "pragmatic  study"[Title/Abstract] OR naturalistic[Title/Abstract] OR "practice setting"[Title/Abstract]  OR "routine care"[Title/Abstract] OR "routine practice"[Title/Abstract] OR "practice-  based"[Title/Abstract] OR "practice based"[Title/Abstract] OR "clinical  outcome"[Title/Abstract] OR "outcomes research"[Title/Abstract] OR  cohort[Title/Abstract] OR cohort study[Title/Abstract] OR retrospective[Title/Abstract]  OR prospective[Title/Abstract] OR "case series"[Title/Abstract] OR "managed  access"[Title/Abstract] OR "early access"[Title/Abstract] OR "expanded  access"[Title/Abstract] OR "cost effectiveness"[Title/Abstract] OR "cost-  effectiveness"[Title/Abstract] OR pharmacovigilance[Title/Abstract] ) |
| Embase | ((difelikefalin or Korsuva or Kapruvia or "CR 845" or CR-845 or CR845 or "kappa opioid  receptor agonist") and ("real-world" or "real world" or "real-world evidence" or "real-  world data" or "real-world use" or RWE or observational or observational study or  observational studies or "real-life" or "real life" or "clinical practice" or "clinical  experience" or "routine clinical practice" or effectiveness or effectiveness study or  registry or registries or "disease registry" or database or databases or "electronic health  record" or "electronic health records" or EHR or claims or "claims data" or "post-  marketing" or "post marketing" or "post-market" or postapproval or post-approval or  "actual use" or pragmatic or "pragmatic study" or naturalistic or "practice setting" or  "routine care" or "routine practice" or "practice-based" or "practice based" or "clinicaloutcome" or "outcomes research" or cohort or cohort study or retrospective or  prospective or "case series" or "managed access" or "early access" or "expanded  access" or "cost effectiveness" or "cost-effectiveness" or pharmacovigilance)).ti,ab. |
| Web of Science | TS=((difelikefalin or korcula or kapraunia or "CR 845" or CR-845 or c1845 or "kappa  opioid receptor agonist") and ("real-world" or "real world" or "real-world evidence" or  "real-world data" or "real-world use" or RWE or observational or observational study or  observational studies or "real-life" or "real life" or "clinical practice" or "clinical  experience" or "routine clinical practice" or effectiveness or effectiveness study or  registry or registries or "disease registry" or database or databases or "electronic health  record" or "electronic health records" or EHR or claims or "claims data" or "post-  marketing" or "post marketing" or "post-market" or postapproval or post-approval or  "actual use" or pragmatic or "pragmatic study" or naturalistic or "practice setting" or  "routine care" or "routine practice" or "practice-based" or "practice based" or "clinical  outcome" or "outcomes research" or cohort or cohort study or retrospective or  prospective or "case series" or "managed access" or "early access" or "expanded  access" or "cost effectiveness" or "cost-effectiveness" or pharmacovigilance)) |
| Scopus | TITLE-ABS-KEY ((difelikefalin or korcula or kapraunia or "CR 845" or CR-845 or c1845 or  "kappa opioid receptor agonist") and ("real-world" or "real world" or "real-world  evidence" or "real-world data" or "real-world use" or RWE or observational or  observational study or observational studies or "real-life" or "real life" or "clinical  practice" or "clinical experience" or "routine clinical practice" or effectiveness or  effectiveness study or registry or registries or "disease registry" or database or  databases or "electronic health record" or "electronic health records" or EHR or claims  or "claims data" or "post-marketing" or "post marketing" or "post-market" or  postapproval or post-approval or "actual use" or pragmatic or "pragmatic study" or  naturalistic or "practice setting" or "routine care" or "routine practice" or "practice-  based" or "practice based" or "clinical outcome" or "outcomes research" or cohort or  cohort study or retrospective or prospective or "case series" or "managed access" or  "early access" or "expanded access" or "cost effectiveness" or "cost-effectiveness" or  pharmacovigilance)) |
| CENTRAL | ((difelikefalin or korcula or kapraunia or "CR 845" or CR-845 or c1845 or "kappa opioid  receptor agonist") and ("real-world" or "real world" or "real-world evidence" or "real-  world data" or "real-world use" or RWE or observational or observational study or  observational studies or "real-life" or "real life" or "clinical practice" or "clinical  experience" or "routine clinical practice" or effectiveness or effectiveness study orregistry or registries or "disease registry" or database or databases or "electronic health  record" or "electronic health records" or EHR or claims or "claims data" or "post-  marketing" or "post marketing" or "post-market" or postapproval or post-approval or  "actual use" or pragmatic or "pragmatic study" or naturalistic or "practice setting" or  "routine care" or "routine practice" or "practice-based" or "practice based" or "clinical  outcome" or "outcomes research" or cohort or cohort study or retrospective or  prospective or "case series" or "managed access" or "early access" or "expanded  access" or "cost effectiveness" or "cost-effectiveness" or pharmacovigilance)):ti,ab,kw |
| CINAHL | ((difelikefalin or korcula or kapraunia or "CR 845" or CR-845 or c1845 or "kappa opioid  receptor agonist") and ("real-world" or "real world" or "real-world evidence" or "real-  world data" or "real-world use" or RWE or observational or observational study or  observational studies or "real-life" or "real life" or "clinical practice" or "clinical  experience" or "routine clinical practice" or effectiveness or effectiveness study or  registry or registries or "disease registry" or database or databases or "electronic health  record" or "electronic health records" or EHR or claims or "claims data" or "post-  marketing" or "post marketing" or "post-market" or postapproval or post-approval or  "actual use" or pragmatic or "pragmatic study" or naturalistic or "practice setting" or  "routine care" or "routine practice" or "practice-based" or "practice based" or "clinical  outcome" or "outcomes research" or cohort or cohort study or retrospective or  prospective or "case series" or "managed access" or "early access" or "expanded  access" or "cost effectiveness" or "cost-effectiveness" or pharmacovigilance)) –  Title/abstract |

**Supplementary Table 3: Study inclusion and exclusion criteria.**

|  | **Inclusion** | **Exclusion** |
| --- | --- | --- |
| **Population** | - Patients with CKD-aP diagnosed according to study criteria - Patients on haemodialysis (all modalities: conventional HD, nocturnal HD, haemodiafiltration) - Patients on peritoneal dialysis (continuous ambulatory peritoneal dialysis [CAPD], automated peritoneal dialysis [APD]) - Patients with advanced non-dialysis CKD (eGFR <30 mL/min/1.73m² or CKD stage 4–5) - Patients post-kidney transplantation with persistent CKD-aP - Any age - All racial and ethnic groups - Any comorbidity profile | - Pruritus attributed to causes other than CKD (e.g., hepatic disease, dermatological conditions, medications) - Studies examining difelikefalin in populations without CKD |
| **Intervention** | Difelikefalin (any formulation, any dose, any regimen) |  |
| **Comparator** | No restriction on comparators; included are:   - Placebo - Standard care/best supportive care - Active comparators (gabapentin, pregabalin, topical agents, other CKD-aP treatments) - Historical controls or baseline-to-follow-up comparisons (single-arm studies) |  |
| **Outcomes** | Primary Outcomes (for meta-analysis consideration):   1. Pruritus Severity Reduction: • Proportion of participants achieving ≥3-point Worst Itch Numerical Rating Scale (WI-NRS) reduction from baseline • Mean change in WI-NRS from baseline • Other severity measures (5-D Itch total score, Skindex-10 total score, visual analogue scale [VAS], verbal rating scale [VRS]) 2. Treatment Safety: • Proportion with any adverse event (AE) • Proportion with serious adverse events (SAEs) • Treatment discontinuation due to adverse events (percentage) • Individual AE frequencies (diarrhoea, nausea, dizziness, gait disturbances, other CNS or gastrointestinal events)   Secondary Outcomes:   3. Quality of Life: • Changes in multidimensional scales (5-D Itch, Skindex-10, KDQOL-36) • Sleep quality (Sleep Quality Numeric Rating Scale, PSQI, or equivalent) • Depression/anxiety (Patient Health Questionnaire [PHQ-9], Generalised Anxiety Disorder [GAD-7], Hospital Anxiety and Depression Scale [HADS]) • Functional outcomes (work productivity, social functioning) 4. Treatment Persistence: • Treatment discontinuation rates (any cause) • Discontinuation rates by reason (AE, lack of efficacy, other) • Median time to discontinuation • Treatment adherence or compliance rates (if reported) 5. Predictors of Response: • Baseline characteristics associated with treatment response (age, sex, race/ethnicity, baseline severity, dialysis vintage, dialysis adequacy parameters, comorbidities) • Subgroup effectiveness analyses 6. Cost-Effectiveness: • Incremental cost-effectiveness ratio (ICER) per quality-adjusted life-year (QALY) • Cost per treatment responder • Budget impact analyses |  |

**Supplementary Table 4: Outcomes for data extraction.**

| **Outcome type** | **Outcomes** |
| --- | --- |
| Study characteristics | Author, year, country, funding source, study duration, registry status |
| Study design | Type (RCT, cohort, case series, registry), prospective/retrospective, comparator presence, blinding |
| Population | Number enrolled and analysed, age (mean, SD, range), sex (% male), race/ethnicity, baseline WI-NRS or other severity measure, dialysis type/modality, dialysis vintage, eGFR (if non-dialysis), comorbidities (diabetes, hypertension, secondary hyperparathyroidism), prior anti-itch treatments |
| Intervention | Dose (mcg/kg), frequency, route (IV), duration of treatment, concomitant medications |
| Outcomes: Pruritus | WI-NRS (baseline, follow-up, change, %responders ≥3-point), 5-D Itch (baseline, follow-up, change), Skindex-10 (baseline, follow-up, change), other severity measures, complete response rates |
| Outcomes: QoL | Sleep quality scores, depression/anxiety measures, functional outcomes, any QoL scales (e.g., SF-36) |
| Outcomes: Safety | AE frequency (any, by type), SAE frequency, discontinuation (total %, by reason), timing of AEs, relationship to study drug |
| Outcomes: Treatment persistence | Discontinuation rates, time to discontinuation, adherence/compliance data |
